# Concomitant Exacerbation of Graves Orbitopathy and Double-Seronegative Myasthenia Gravis after SARS-CoV-2 Infection

**DOI:** 10.1210/jcemcr/luaf019

**Published:** 2025-01-29

**Authors:** Yuto Nakano, Ken Takeshima, Yasushi Furukawa, Shuhei Morita, Mayumi Sakata, Taka-Aki Matsuoka

**Affiliations:** First Department of Internal Medicine, Wakayama Medical University, Wakayama 641-8509, Japan; First Department of Internal Medicine, Wakayama Medical University, Wakayama 641-8509, Japan; First Department of Internal Medicine, Wakayama Medical University, Wakayama 641-8509, Japan; First Department of Internal Medicine, Wakayama Medical University, Wakayama 641-8509, Japan; Department of Neurology, Wakayama Medical University, Wakayama 641-8509, Japan; First Department of Internal Medicine, Wakayama Medical University, Wakayama 641-8509, Japan

**Keywords:** SARS-CoV-2, Graves orbitopathy, myasthenia gravis, anti-AChR antibody, anti-MuSK antibody, Th17

## Abstract

SARS-CoV-2 infection could trigger autoimmune disease. We report a case of concomitant exacerbation of Graves orbitopathy (GO) and myasthenia gravis (MG) after SARS-CoV-2 infection. A 43-year-old woman had diplopia, proptosis, and swollen eyelids. Blood tests showed thyrotoxicosis and positive thyroid-stimulating hormone receptor antibodies, and orbital magnetic resonance imaging (MRI) showed enlarged extraocular muscles. She was therefore referred to our hospital with diagnosis of GO. Methylprednisolone pulse therapy (MPT) in combination with orbital radiotherapy were performed for 3 weeks, and ocular symptoms improved. At 41 weeks, the patient was infected with SARS-CoV-2 and felt sudden worsening of diplopia and ptosis. MRI showed an enlarged right inferior rectus muscle. MPT and orbital radiotherapy were performed again for 3 weeks for the suspected GO, but there was insufficient improvement of the ptosis. Serum antiacetylcholine receptor and anti–muscle-specific tyrosine kinase antibodies were negative, but the patient was further evaluated with repetitive nerve stimulation test and ice pack test, and diagnosis was double-seronegative MG. Pyridostigmine treatment led to dramatic improvement of the ptosis. SARS-CoV-2 infection could exacerbate MG as well as GO. Careful diagnosis is required for ocular symptoms after SARS-CoV-2 infection, especially when there is double-seronegative MG.

## Introduction

Excessive immune responses can trigger a systemic cytokine storm in COVID-19, leading to multiple organ failure [1, 2]. Although the fatality rate of COVID-19 is decreasing, there are reports of exacerbation of autoimmune diseases after SARS-CoV-2 infection [3].

Autoimmune thyroid diseases, such as subacute thyroiditis, painless thyroiditis, and Graves disease (with or without orbitopathy), have been reported after SARS-CoV-2 infection [4, 5]. Myasthenia gravis (MG), an autoimmune disorder affecting neuromuscular transmission, has also been reported in association with SARS-CoV-2 infection [6]. Simultaneous exacerbation of Graves orbitopathy (GO) and MG after SARS-CoV-2 infection is extremely rare, and the causal relationship is still unclear.

## Case Presentation

A 46-year-old woman had eyelid swelling, diplopia, and difficulty opening her eyes, and was referred to our hospital for further evaluation. She had no family history of thyroid disease, but was sensitive to heat and felt excessive sweating for approximately 2 years. She smoked 15 cigarettes per day.

**Figure 1.**
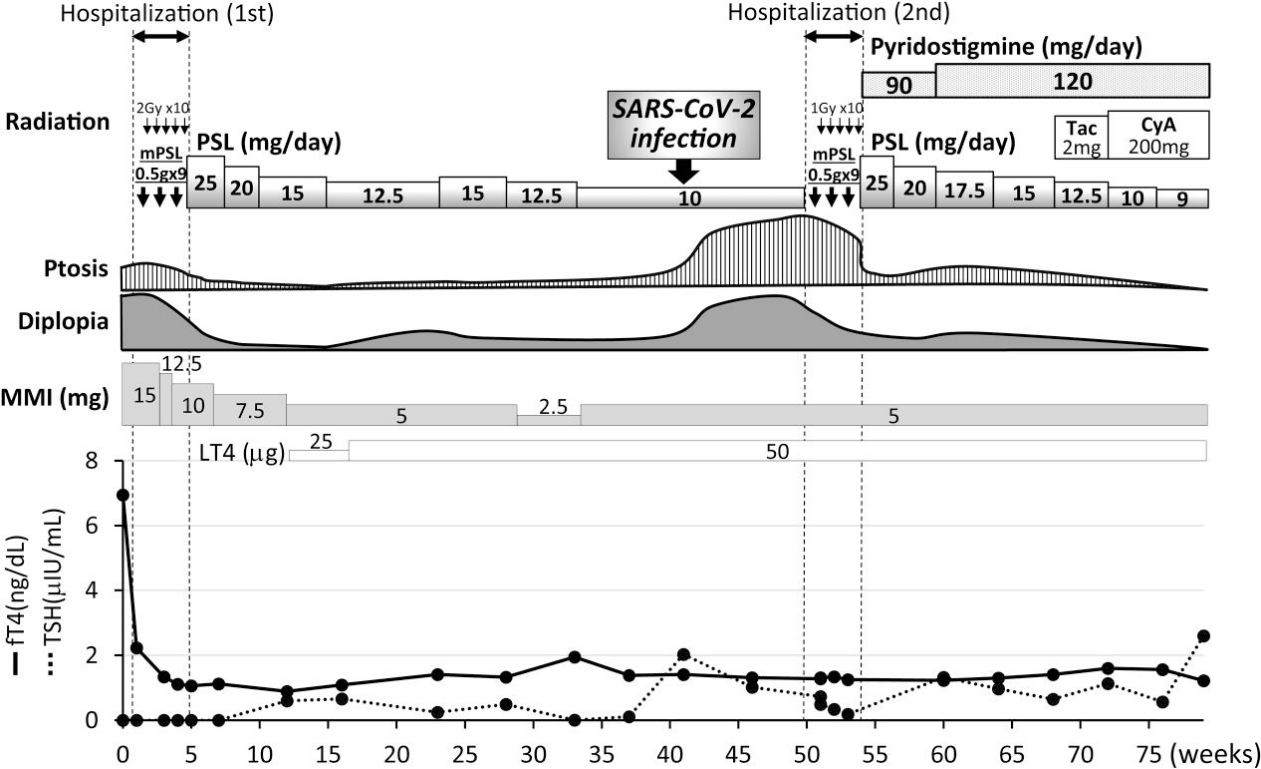
Clinical course of Graves disease and orbitopathy. On first admission, diplopia improved dramatically after methylprednisolone pulse therapy and orbital radiotherapy. Ocular symptoms reexacerbated after SARS-CoV-2 infection. On second admission, pyridostigmine was quite effective for the ptosis, despite the limited effects of the combination therapy for Graves orbitopathy. CyA, cyclophosphamide; fT4, free thyroxine; LT4, levothyroxine; MMI, methimazole; mPSL, methylprednisolone; PSL, prednisolone; Tac, tacrolimus; TSH, thyrotropin.

**Figure 2.**
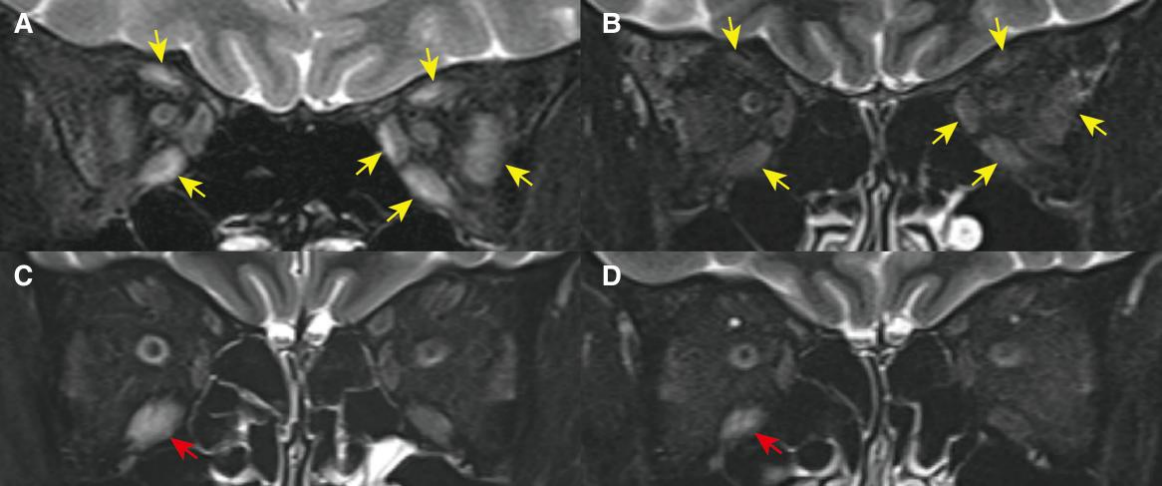
Short tau inversion recovery images of orbital magnetic resonance imaging during treatment for Graves orbitopathy. Enlarged extraocular muscles with high signal intensities (A, yellow arrows) improved after treatment with steroid pulse therapy and ocular radiotherapy (B, yellow arrows) on first admission. Enlargement of the right inferior rectus muscle with high signal intensities after SARS-CoV-2 infection (C, a red arrow), which improved after the combination therapy (D, a red arrow) on second admission.

Neck palpation revealed a diffuse elastic soft goiter. She had bulging eyes and her eyelids were swollen with conjunctival hyperemia. An objective eye movement test revealed binocular ocular motility disorder in all directions. She felt no diurnal variation of the ocular symptoms. Blood tests showed thyrotoxicosis and high titers of anti–thyrotropin receptor antibody (TRAb) and anti-thyrotropin antibody (TSAb), and negative anti-acetylcholine receptor (AChR) antibodies (Table 1). We therefore diagnosed Graves disease and orbitopathy and started methimazole 15 mg/day (Fig. 1). Magnetic resonance imaging (MRI) revealed bilateral enlargement of the extraocular muscles, and short tau inversion recovery (STIR) images showed high signal intensities of the muscles, suggesting active inflammation (Fig. 2A). Clinical activity score was 7 of 10 points, so she was admitted as an inpatient for intravenous methylprednisolone pulse therapy (MPT) and orbital radiotherapy for GO. Hyperthyroidism rapidly improved after starting methimazole, the diplopia and swollen eyelids markedly improved, and the clinical activity score decreased to 2 of 10 points within 6 weeks after starting the combination therapy (see Fig. 1). Oral prednisolone (PSL) 25 mg/day was started after MPT and gradually reduced. The patient was discharged from our hospital after 3 weeks.

**Table 1.** Laboratory data associated with Graves disease and myasthenia gravis on first and second hospitalizations

	Reference range	First hospitalization	Second hospitalization
TSH	0.61-4.23 µIU/mL (0.61-4.23 mIU/L)	<0.003 µIU/mL (<0.003 mIU/L)	0.73 µIU/mL (0.73 mIU/L)
fT4	0.90-1.70 ng/dL (11.5-21.8 pmol/L)	6.95 ng/dL (89.4 pmol/L)	1.28 ng/dL (16.4 pmol/L)
fT3	2.30-4.00 pg/mL (3.53-6.14 pmol/L)	17.0 pg/mL (26.11 pmol/L)	2.05 pg/mL (3.15 pmol/L)
TRAb	<2.0 IU/L	60.2 IU/L	ND
TSAb	<120%	4950%	917%
anti-AChR Ab	≤0.3 nmol/L	≤0.3 nmol/L	≤0.3 nmol/L
anti-Musk Ab	<0.02 nmol/L	ND	<0.01 nmol/L

Abbreviations: Ab, antibody; AChR, acetylcholine receptor; fT3, free triiodothyronine; fT4, free thyroxine; MuSK, muscle-specific tyrosine kinase; ND, no data; TRAb, thyrotropin receptor antibody; TSAb, thyrotropin antibody; TSH, thyrotropin.

Thirty-six weeks later, the patient had a fever and sore throat and visited a nearby hospital. SARS-CoV-2 nasal antigen test and nasopharyngeal reverse-transcription polymerase chain reaction test revealed infection with SARS-CoV-2. After recovery from typical respiratory symptoms associated with COVID-19, diplopia and ptosis dramatically worsened, especially on the right side. She was therefore readmitted 2 weeks later to our hospital to treat suspected GO.

## Diagnostic Assessment

On the patient's initial admission, blood tests had shown thyrotoxicosis with elevated TRAb and TSAb levels (see Table 1). Orbital MRI had shown enlarged bilateral extraocular muscles with high signal intensity on STIR image (Fig. 2A). These abnormal MRI findings improved after combination treatment with MPT and orbital radiotherapy (Fig. 2B).

On her second admission, blood tests showed that she was euthyroid and TSAb levels were 917% (see Table 1). Orbital MRI showed predominant enlargement of right inferior rectus muscles with high signal intensity on STIR image (Fig. 2C), compatible with the exacerbation of GO. The second course of MPT and orbital radiotherapy was effective for the enlargement and inflammatory MRI findings of the ocular muscles (Fig. 2D), but the patient reported continued ptosis, which was especially enhanced in the afternoon. Diurnal fluctuation of the symptoms is characteristic of MG, so we evaluated anti–muscle-specific tyrosine kinase (MuSK) antibodies and anti-AChR antibodies: Both were negative (see Table 1).

To further evaluate for a neuromuscular junction disorder, we performed a repetitive nerve stimulation test, which revealed waning at low frequent repetitive stimulation, and the ice pack test, which showed improvement of ptosis. These results were consistent with MG. The patient had no impairment of limb or respiratory muscles, so we diagnosed concomitant ocular MG.

## Treatment

A second round of MPT treatment was followed by oral PSL 25 mg/day (see Fig. 1). In addition, the patient was started on pyridostigmine bromide 90 mg/day for concomitant MG.

## Outcome and Follow-up

**Figure 3.**
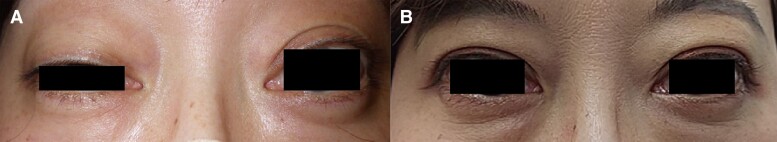
The effect of pyridostigmine on ptosis. Ptosis improved dramatically after treatment with pyridostigmine. A, before treatment; B, after treatment.

The patient felt immediate improvement of ptosis after starting pyridostigmine, and her diplopia gradually improved (Fig. 3). She sometimes felt slight worsening of diplopia and ptosis when she was fatigued, so pyridostigmine was increased to 120 mg/day after 7 weeks (see Fig. 1).

PSL was tapered to 12.5 mg/day after the addition of tacrolimus 2 mg/day. After 4 weeks, however, tacrolimus was changed to cyclophosphamide 200 mg/day due to nausea and diarrhea. At 76 weeks, PSL was successfully reduced to 9 mg/day with satisfactory control of the ocular symptoms associated with GO and MG.

## Discussion

GO with MG may be exacerbated after SARS-CoV-2 infection. On our patient's first admission, she presented with severe diplopia and orbital MRI showed increased STIR signal intensity of swollen extraocular muscles. Her ocular symptoms improved dramatically after MPT and orbital radiotherapy—a typical clinical course of GO. On readmission, the combination therapy was effective for the enlarged ocular muscles, but had limited effects on the ptosis, which had diurnal fluctuation. Pyridostigmine was quite effective for the ptosis, so her ocular symptoms (especially ptosis) were thought to be mainly due to MG. Establishing the diagnosis of MG was difficult because the ocular symptoms overlapped with GO and because both anti-AChR and anti-MuSK antibodies were negative, so-called “double seronegative MG.”

Association between SARS-CoV-2 infection and development or exacerbation of several thyroid diseases have been reported. For example, Brancatella et al first reported a case of subacute thyroiditis after SARS-CoV-2 infection, and then several similar case series [7, 8]. An association between Graves disease and SARS-CoV-2 infection has also been reported [4, 5, 9, 10]. According to a systematic review of 20 cases with autoimmune thyroiditis after SARS-CoV-2 infection, 14 cases were Graves disease, 5 cases were hypothyroidism due to Hashimoto thyroiditis, and 1 case was postpartum thyroiditis [5]. Two patients with Graves disease reportedly developed GO, which was also shown in the present case. The first case was that of a woman who had been treated for Graves disease controlled with carbimazole [10]. Her thyroid function worsened after SARS-CoV-2 infection, and mild proptosis and swollen eyelids developed. The second case was that of a woman who developed hyperthyroidism 2 months after SARS-CoV-2 infection and was diagnosed for the first time with Graves disease [9]. She also had mild GO with conjunctival hyperemia, but had no proptosis or ocular motility disorders. Our patient's clinical course differs from these two cases in that she had worsening of previously treated GO, but not of thyroid function after SARS-CoV-2 infection, and that her ocular manifestations justified the need for MPT.

Overlapping GO and MG following SARS-CoV-2 infection has only once been previously reported [11]. A 74-year-old Italian man presented with severe diplopia 1 month after SARS-CoV-2 infection, with bilateral eye protrusion and disturbance of eyelid elevation, ocular motility disorder, and fatigue. Elevated serum levels of TRAb and anti-AChR antibody led to diagnosis of Graves disease with orbitopathy and concomitant MG. The severity of GO was mild and his symptoms were predominantly due to MG, so ocular symptoms improved after treatment with oral PSL 10 mg/day and pyridostigmine 90 mg/day. Notably, our patient's ocular symptoms developed in 2 phases: 1 before SARS-CoV-2 infection that was mainly due to GO with marked response to MPT, and another after SARS-CoV-2 infection mainly due to MG with marked response to pyridostigmine but limited response to MPT.

**Figure 4.**
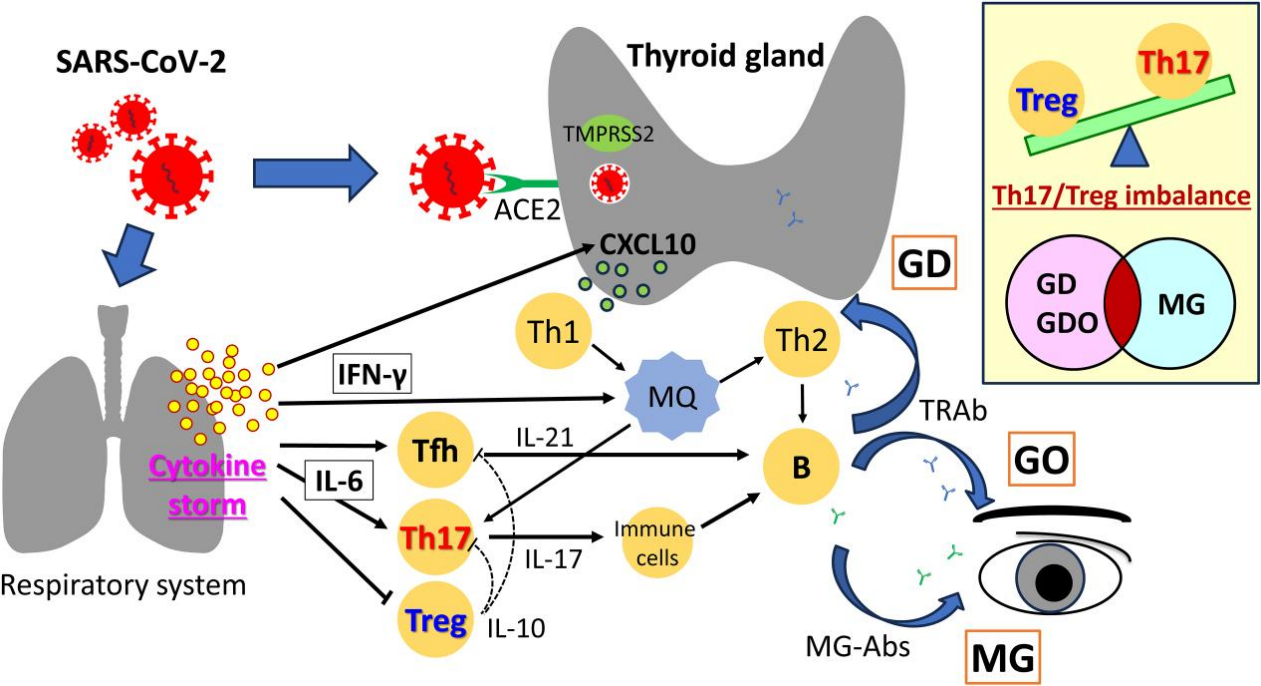
Postulated causes of worsened Graves orbitopathy and myasthenia gravis following SARS-CoV-2 infection. SARS-CoV-2 infection induces cytokine storm and an imbalance between Th17 and Treg as well as Tfh activation, followed by an excessive autoimmune response. Ab, antibody; ACE2, angiotensin-converting enzyme; APRIL, a proliferation-inducing ligand; BAFF, B cell–activating factor; CXCL10, C-X-C motif chemokine ligand 10; GD, Graves disease; GO, Graves orbitopathy; IFN, interferon; IL, interleukin; MG, myasthenia gravis; MQ, macrophage; Tfh, follicular T cell; Th, helper T cell; TMPRSS2, transmembrane protease serine 2; Treg, regulatory T cell.

As for the effect of SARS-CoV-2 infection on the thyroid, various mechanisms have been proposed (Fig. 4). First, direct infection of SARS-CoV-2 on thyroid follicular cells has been suggested. SARS-CoV-2 reportedly uses spike proteins and angiotensin-converting enzyme 2 (ACE2) receptor for entry and transmembrane protease serine 2 (TMPRSS2) for S protein cleavage, followed by intrusion to the host cells [12]. ACE2 and TMPRSS2 are both known to be highly expressed in the thyroid as in the lungs, so direct infection of a virus into thyroid follicular cells could occur [13]. The pathogenesis of subacute thyroiditis and painless thyroiditis may therefore be initiated from the apoptosis of virus-infected cells, followed by presentation of thyroid-derived autoantigens, leading to follicular cell destruction by cytotoxic T cells [14].

Second, cytokine storms caused by SARS-CoV-2 infection could indirectly modulate autoimmunity associated with thyroid diseases (see Fig. 4). Inflammatory cytokines such as interferon (IFN) γ and interleukin (IL)-6 elevate in the state of cytokine storms. IFN₋γ induces helper T cell (Th) 1 in the thyroid and activates macrophages via increased expression of C-X-C motif chemokine ligand 10 (CXCL10), a cytokine elevated in the active phase of Graves disease, in thyroid cells [15]. Autoantigens are presented by the activated macrophages, and autoantibodies such as TRAb are produced by activation of Th2 and B cells. Conversely, IL-6 activates follicular helper T cell (Tfh) and Th17 while suppressing regulatory T cells (Tregs) [14]. In patients with Graves disease, Tfh2 (a subset of Tfh) is reportedly elevated and contributes to the differentiation of B cells to plasma cells [16]. Th17 activates immune cells and is associated with B-cell maturation and survival, which is important in producing antibodies in Graves disease [17]. Tregs are responsible for suppressing Tfh and Th17 and the reduction of Tregs has been reported in patients with Graves disease and GO [18]. An imbalance between Th17 and Treg caused by SARS-CoV-2 infection may therefore play an important role in the development and exacerbation of Graves disease and GO.

New-onset or worsening of MG after SARS-CoV-2 infection has also been reported. In a review of 18 cases of new-onset MG following SARS-CoV-2 infection, 2 patients had Hashimoto thyroiditis and 1 patient had Graves disease; 16 were positive for anti-AChR antibodies and 2 for anti-MuSK antibodies [6]. Unlike in the present case, none of the cases had double-seronegative MG.

As for the cytokines in patients with MG, Th17 cells and their associated cytokines are reportedly increased, while the Tregs are decreased in the peripheral blood cells [19-21]. Correlation between the frequency of Th17 cells and anti-AChR titers has also been reported [19]. Treg cell counts reportedly increased after treatment with immunosuppressants and were inversely correlated with clinical manifestations in patients with MG [21].

An imbalance between Th17 and Treg cells could be a common pathophysiology both in Graves disease and MG. We therefore speculate as to the mechanism that occurred in our patient (see Fig. 4): SARS-CoV-2 infection triggered a cytokine storm and caused an imbalance between Th17 and Tregs as well as the activation of Tfh. We think this led to an excessive autoimmune response followed by exacerbation of GO and MG.

SARS-CoV-2 infection may exacerbate both GO and MG based on their common underlying pathophysiology. Careful differential diagnosis of ocular manifestations after SARS-CoV-2 infection is therefore required.

## Learning Points

- SARS-CoV-2 infection may exacerbate both GO and MG.
- If patients with GO feel worsening of ocular symptoms after SARS-CoV-2 infection, MG should be carefully excluded despite negativity for both anti-AChR and anti-MuSK antibodies.
- Imbalance between Th17 and Treg cells could be a common underlying pathophysiology associated with exacerbation both of GO and MG after SARS-CoV-2 infection.

## Data Availability

Data sharing is not applicable to this article as no data sets were generated or analyzed during the present study.

## Abbreviations

ACE2: angiotensin-converting enzyme 2
AChR: acetylcholine receptor
GO: Graves orbitopathy
IFN: interferon
IL: interleukin
MG: myasthenia gravis
MPT: methylprednisolone pulse therapy
MRI: magnetic resonance imaging
MuSK: muscle-specific tyrosine kinase
PSL: prednisolone
STIR: short tau inversion recovery
Tfh: follicular helper T cell
Th: helper T cell
TRAb: thyrotropin receptor antibody
Treg: regulatory T cell
TSAb: thyrotropin antibody
